# A novel minimally invasive technique of inter-spinal distraction fusion surgery for single-level lumbar spinal stenosis in octogenarians: a retrospective cohort study

**DOI:** 10.1186/s13018-022-03004-9

**Published:** 2022-02-16

**Authors:** Mengmeng Chen, Pu Jia, Fei Feng, Hai Tang

**Affiliations:** Department of Orthopaedics, Beijing Friendship Hospital, Capital Medical University, Beijing, No. 95, Yong An Road, Xi Cheng District, Beijing, 100050 People’s Republic of China

**Keywords:** Lumbar spinal stenosis, Octogenarians, Inter-spinal distraction fusion, Posterior lumbar interbody fusion, Clinical efficacy

## Abstract

**Objective:**

Surgical treatment of lumbar spinal stenosis (LSS) in octogenarians (patients aged ≥ 80 years) has been a challenge. Inter-spinal distraction fusion (ISDF)—a minimally invasive procedure—was used for treating LSS in octogenarians. This retrospective cohort study aimed to investigate the clinical efficacy and safety of a minimally invasive ISDF technique for LSS in octogenarian patients.

**Methods:**

From April 2015 to April 2019, octogenarian patients who underwent lumbar fusion surgery due to single-segment LSS were included. The patients were grouped into the ISDF group and posterior lumbar interbody fusion (PLIF) group based on the type of surgery. Clinical outcomes were evaluated using scores of the visual analog pain scale (VAS), Oswestry Disability Index (ODI), and Japanese Orthopedics Association (JOA) scale. Radiographs were assessed for the intervertebral angle (IA), lumbar lordosis (LL), and posterior disc height (PDH). After 2 years postoperatively, all patients underwent computed tomography (CT) to evaluate the fusion condition. Perioperative data and related complications were recorded.

**Results:**

Sixty-two patients were included (mean age: 82.22 ± 1.95 years). The ISDF and the PLIF groups had 34 and 28 patients, respectively. The average follow-up time was 2.1 ± 0.25 years. There was no significant difference in VAS, ODI, JOA, and PDH scores between both groups preoperatively and at each postoperative time-point. The IA and LL showed significant differences between both groups after surgery (*p* < 0.05). The postoperative IA in the ISDF group were significantly lower than the preoperative values, while that in the PLIF group were markedly increased. The PLIF group had an increased LL compared with that preoperatively (*p* < 0.05), while the LL in the ISDF did not significantly change. The operative time, blood loss, hospital stay time, and the rate of perioperative complications of the ISDF group were significantly lower than those of the PLIF group (*p* < 0.05). There was no significant difference in the fusion rates between both groups.

**Conclusion:**

ISDF surgery is a viable method for octogenarian patients with LSS that provides a similar clinical efficacy, shorter operative time, less blood loss, shorter hospital stay time, and fewer complications, compared to the PLIF surgery.

## Introduction

As medical technologies have advanced and public health establishments underwent improvements, longevity rates increased and led to the gradual increase in the population of elderly people in some industrial cities[1]. Octogenarians (aged ≥ 80 years) comprise the fastest growing age group globally, which causes an increase in the incidence of age-related diseases, including lumbar disc herniation, stenosis, and spondylolisthesis[2]. Lumbar spinal stenosis (LSS) is a common age-related degenerative disease affecting the mobility of elderly persons with the classic characteristic of intermittent claudication[3]. Treatment options include conservative management (drug therapy, physiotherapy, or epidural steroid injections) and surgical intervention. Patients with persistent symptoms, and conservative management fails to show improvement require surgery[4]. However, many octogenarian patients have other coexisting conditions that involve multiple systems[5]. The complexities of the health status increase the risk of surgery. Therefore, for the management of octogenarian patients who require surgical decompression, a suitable surgical regimen needs to be developed after weighing the pros and cons.

Some investigators reported that a favorable clinical efficacy can be obtained with decompression alone in octogenarians[6, 7]. However, extensive decompression may increase the risk of potential spinal instability. Moreover, a considerable proportion of patients with LSS have other forms of degeneration (disc degeneration, spondylolisthesis, and joint facet hyperplasia), which simple decompression cannot resolve. Posterior lumbar interbody fusion (PLIF) instrumented with pedicle screws is a classical treatment for lumbar degenerative diseases[8]; however, larger trauma and longer operation times are associated with PLIF, which lead to perioperative complications. Most octogenarians cannot withstand traditional lumbar interbody fusion procedures. It has been reported that the complication rates can reach 71.4% for octogenarians undergoing traditional lumbar fusion surgeries, and the 1-year mortality rate is 4.2%[9]. Meanwhile, adjacent segmental degeneration caused by interbody fusion provides little chance for performing revision surgeries in octogenarians[10]. Such disadvantages of PLIF induces fear regarding the uncertain postoperative consequences, which forces many octogenarians to tolerate prolonged suffering without undergoing surgical treatment. This severely influences their quality of life.

The concept of minimally invasive surgery has become prominent in the recent past, which has enabled the use of inter-spinal distraction fusion (ISDF) for treating lumbar degenerative disease. In fact, Spadea et al. reported the use of an interspinous fusion surgery to treat lumbar disc herniation in 1952. Since reliable implants were lacking at that time, they resected the L3 spinous process as a bone graft material to implant the L4–L5 interspinous space. Definitive fusion of the interspinous process was observed in several cases of revision surgery after several years[11]. In recent years, advanced interspinous devices have been developed to satisfy different clinical requirements. The ISDF device can be used to distract the interspinous space, stabilize the decompressed segment, and finally achieve interspinous fusion[12, 13]. The ISDF device can be firmly fixed on the spinous process with spikes on the lateral plate to provide stability[14, 15] and the hollow space in the device can be filled with a bone graft material and bone autograft to enable interspinous fusion[16]. Therefore, it is a kind of fused interspinous stabilization device.

ISDF provides advantages including less damage to the tissue structures, shorter operative time, and early ambulation, which are useful for lumbar disc herniation, LSS, and mild degenerative spondylolisthesis[17–20]. Wei et al. treated lumbar disc herniation by ISDF surgery and demonstrated a 92.6% successful outcome in a retrospective study[17]. Sclafani et al. managed lumbar degenerative diseases with a polyaxial ISDF device and reported satisfactory clinical efficacy[18]. ISDF is especially suitable for geriatric patients with several comorbidities and a higher risk of intolerance to screw-rod surgery[19]. However, there are no reports on the use of ISDF in octogenarian patients. Therefore, we designed a retrospective study to investigate the clinical and radiological outcomes of LSS in octogenarians treated using ISDF and compare the outcomes with those of traditional PLIF.

## Methods

This retrospective study was approved by the Bioethics Committee of the Beijing Friendship Hospital. The need for informed consent was waived considering that no patient identification information was used.

### Patients

Octogenarian patients with LSS who were referred to our hospital between April 2015 and April 2019 were enrolled. The inclusion criteria were as follows: (1) age ≥ 80 years; (2) diagnosis of single-segment LSS confirmed by computed tomography (CT) or magnetic resonance imaging (MRI); (3) lower extremity neurologic symptoms with or without intermittent claudication; (4) undergoing PLIF or ISDF surgery; and (5) follow-up period ≥ 2 years. The exclusion criteria were as follows: (1) Meyerding II degenerative spondylolisthesis or above; (2) lumbar instability; (3) posterior arch weakness or deficiency; (4) history of spinal surgery, trauma, or tumor; and (5) severe nervous system disease or peripheral vascular disease. We included 62 patients who met these criteria and were grouped as the ISDF and PLIF groups. All baseline information including age, sex, bone mineral density (BMD), symptom duration, stenosis level, stenosis severity, comorbidities, and American Society of Anesthesiologists (ASA) physical status class were recorded. Comorbidities were assessed using the Charlson comorbidity index (CCI)[21].

#### Surgical technique (ISDF)

After undergoing spinal anesthesia, the patients were placed in the prone position. The surgical site was located using X-ray fluoroscopy. Thereafter, a posterior midline incision was performed to expose the spinous process and lamina of the surgical segment. The supraspinous ligaments kept intact. A soft tissue rasp was inserted into the interspinous space to pierce through the interspinous ligament. Various specifications of dilator were used to gradually expand the interspinous space and determine the size of the ISDF device (BacFuse, Pioneer Surgical Technology Inc., Marquette, MI, US). Decortication of the interspinous process was performed using a bone file to prepare the bone graft bed. Partial laminectomy or facetectomy was performed, and the facet joint was retained to the maximum possible extent on the basis of radical decompression. The thickened ligamentum flavum was removed to fully release the nerve root. The hollow part of the device was filled with a mixture of autogenous bone and bone graft material. The main lateral plate of device was placed from one side of the spinous process using a sleeve. After main lateral plate was inserted into the interspinous space, the sleeve was removed and the other lateral plate was placed form the opposite side. Thereafter, the two lateral plates were closely fixed on the spinous processes with a pressure clamp. Finally, the nut was tightened for locking. The position of internal fixation was confirmed by fluoroscopy, and the incision was sutured.

#### Surgical technique (PLIF)

Patients of the other group underwent surgery in the prone position after general anesthesia was induced. A midline incision by the posterior approach was performed to expose the bilateral laminae and facet joints. Pedicle screws were placed into the upper and lower vertebral pedicles of surgical segment. Partial laminectomy or facetectomy was performed depending on the symptoms, and the ligamentum flavum was removed simultaneously. The nucleus pulposus tissue was removed, and the cartilaginous endplates of the upper and lower vertebral bodies were scraped off using a curette. The intervertebral space was filled with the prepared autograft and inserted with interbody fusion cage. Longitudinal compression was performed to ensure a tight contact with the endplates of the upper and lower vertebral bodies. Two rods were then placed on the bilateral screws and tightened, and a crosslink was used to connect the bilateral rods. Finally, the screw rod positioning was checked by fluoroscopy, and the nut was fixed firmly. A drainage tube was placed, and the incision was sutured in the anatomical layers.

### Clinical evaluation

All data of the included patients were recorded at the preoperative, postoperative, 6 months, and 2 years follow-up time points. The visual analog scale (VAS) was used to evaluate pain conditions[22]. The Oswestry Disability Index (ODI) and Japanese Orthopedics Association (JOA) scale were used to assess the clinical function status[23, 24]. (1) Leg pain was assessed on a VAS scale of 0–10 (0 representing pain-free and 10 representing the worst pain). (2) The ODI score included ten aspects, such as pain degree and self-caring ability in daily life. For each question, a score of 0–5 was selected for each patient. The calculations were performed using the formula: the actual score/highest possible score × 100%, with higher scores indicating more severe dysfunction[23]. (3) The JOA score included symptoms, signs, daily life, and bladder function. The perfect score was 29. The scores were classified as poor (0–9), moderate (10–15), good (16–24) and excellent (25–29)[24].

We analyzed the operative time, blood loss, blood transfusion, length of hospital stay, and perioperative complications. We further analyzed the occurrence of major complications that were life-threatening or those that could influence the treatment protocols and outcomes, including neurologic injury, epidural hematoma, wound infection, pneumonia, new-onset cardiac arrhythmia, myocardial infarction, stroke, and thromboembolic disease[25].

#### Radiological analysis

The plain radiographs in the anteroposterior and lateral positions were taken at preoperative, postoperative, 6 months and 2 years follow-up time points. Radiological parameters, including intervertebral angle (IA), lumbar lordosis (LL), and posterior disc height (PDH), were assessed in standing neutral lateral radiographs. The IA was measured as the angle between the superior endplate of the upper vertebra and the inferior endplate of the lower vertebra; LL was assessed as the angle between the L1 and S1 superior endplates; PDH was defined as the distance between the posteroinferior margin of the upper vertebra and the posterosuperior margin of the lower vertebra. All included patients underwent CT to evaluate the bony fusion after 2 postoperative years. The formation of a bone bridge between the upper and lower vertebrae was considered a true fusion.

### Statistical analysis

SPSS software (version 20.0; IBM Corp., Armonk, NY, USA) was used for statistical analysis. Measured data are expressed as means ± standard deviation. Data comparisons between preoperative and postoperative timepoints were analyzed using a paired-sample t-test. The comparisons between both groups were evaluated using an independent sample t-test. Chi-square test was used to compare the discontinuous variables of both groups. All data statistics were conducted using a bilateral test, and statistical significance was set at *p* < 0.05.

## Results

### Patient characteristics

The ISDF and PLIF groups had 34 and 28 patients each (age: 82.22 ± 1.95 years; range: 80–87 years). The CCI and ASA were 1.90 ± 0.76 and 2.81 ± 0.40, respectively. The follow-up time was 2.1 ± 0.25 years. Almost every patient had at least one chronic age-related comorbidity, such as hypertension, diabetes mellitus, and coronary heart disease. There were no significant differences in the age, sex, BMD, symptom duration, CCI, ASA, stenosis degree, lesion segment, preoperative VAS, preoperative ODI, and preoperative JOA between both groups (Table 1).

**Table 1:** Preoperative data of two groups

	ISDF	PLIF	*p*
No	34	28	
Age (mean)	82.29 ± 1.9	82.14 ± 2.05	0.764
Gender			0.302
Female	20	20	
Male	14	8	
BMD (SD)	− 1.67 ± 0.92	− 1.5 ± 0.72	0.429
Duration (years)	3.11 ± 1.23	3.09 ± 1.16	0.949
CCI	1.91 ± 0.87	1.89 ± 0.63	0.587
ASA	2.85 ± 0.36	2.68 ± 0.48	0.315
Stenosis degree			0.257
Moderate	23	15	
Severe	11	13	
Treatment level			0.673
L1/2	1	1	
L2/3	2	3	
L3/4	3	1	
L4/5	21	15	
L5/S1	7	8	
Pre-VAS	7.32 ± 0.91	7.18 ± 0.90	0.534
Pre-ODI	51.66 ± 10.74	49.29 ± 9.63	0.367
Pre-JOA	11.56 ± 3.40	11.68 ± 3.10	0.886

### Surgical characteristics and complications

The operative time, blood loss, and hospital stay time of the ISDF group were significantly less than those in the PLIF group (*p* < 0.05) (Table 2). In the ISDF group, all surgeries were completed within 90 min with a blood loss of 46.47 ± 26.27 ml. The blood transfusion rate in the ISDF group was significantly lower than that in the PLIF group (*p* < 0.05). No severe intraoperative complications occurred in either group, such as nerve root damage, major bleeding, or cerebrospinal fluid leakage. However, the major postoperative complication rate in the ISDF group was significantly lower than that in the PLIF group (*p* < 0.05) (Table 3).

**Table 2:** Perioperative parameters

	ISDF	PLIF	*p*
Operative time (min)	76.06 ± 17.31	155.71. ± 61.88	< 0.05
Blood loss (ml)	46.47 ± 26.27	219.29 ± 149.50	< 0.05
Transfusion rate (%)	2.94 (1/34)	46.43 (13/28)	< 0.05
Hospital stay(days)	10.35 ± 3.26	17.39 ± 9.13	< 0.05
Fusion rate (%)	79.41 (27/34)	85.71 (25/28)	0.481
Complications rate (%)	5.88 (2/34)	28.57 (8/28)	0.001

**Table 3:** Complications

	ISDF	PLIF
Epidural hematoma	0	1
Wound infection	1	2
Pneumonia	1	2
Myocardial infarction	0	1
Stroke	0	1
Gastrointestinal hemorrhage	0	1
Total	2	8

### Clinical evaluations

**Fig. 1:**
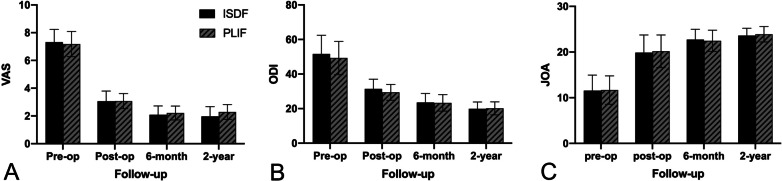
Clinical pain and function indicators at preoperative and each postoperative follow-up point. There was no significant difference between the two groups in VAS, ODI, and JOA. **a** VAS. **b** ODI. **c** JOA. (VAS, visual analogue scale; ODI, Oswestry disability score; JOA, Japanese orthopedics association score)

Compared with preoperative data, the postoperative pain and function indicators showed significant changes in the two groups (Fig. 1). The VAS significantly reduced from preoperative 7.32 ± 0.91 to postoperative 3.05 ± 0.74 in the ISDF group (*p* < 0.05). After 2 years postoperatively, the VAS score was maintained at 1.97 ± 0.7, which was significantly different from the preoperative VAS score (*p* < 0.05). In the PLIF group, it significantly reduced from preoperative 7.18 ± 0.9 to postoperative 3.07 ± 0.54 (*p* < 0.05). After 2 years postoperatively, the VAS was still maintained 2.29 ± 0.53, which was significantly different from that preoperatively (*p* < 0.05). The VAS scores had no significant differences between both groups at any postoperative time point.

The preoperative ODI was 51.66 ± 10.74 in the ISDF group and 49.29 ± 9.63 in the PLIF group. The postoperative ODI scores dropped to 31.42 ± 5.58 in the ISDF group and 29.43 ± 4.57 in the PLIF group (*p* < 0.05). At the 2-year follow-up, the scores were 19.91 ± 3.95 in the ISDF group and 20.14 ± 3.78 in the PLIF group. There was no significant difference in the ODI between the two groups at any postoperative time point.

The preoperative JOA was 11.56 ± 3.40 in the ISDF group and 11.68 ± 3.10 in the PLIF group. The postoperative JOA scores increased to 19.88 ± 3.85 in the ISDF group and 20.18 ± 3.56 in the PLIF group (*p* < 0.05). At the 2-year follow-up, the scores remained at 23.62 ± 1.60 in the ISDF group and 23.89 ± 1.69 in the PLIF group. There was no significant difference in the JOA between the two groups at any postoperative time point.

### Radiological outcomes

**Fig. 2:**
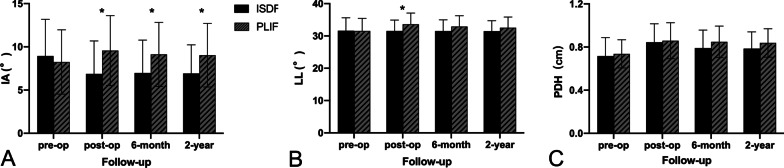
Radiological indicators at preoperative and each postoperative follow-up point. **a** IA. **b** LL. C. PDH. * represented significant difference between the two groups. (IA, intervertebral angle; LL, lumbar lordosis; PDH, posterior disc height)

Figure 2 displays the change in the radiological parameters. In the ISDF group, a significant decrease in IA was observed in the postoperative (6.87 ± 3.81)° outcomes compared to the preoperative values (8.94 ± 4.24)° (*p* < 0.05). The IA was maintained at (6.92 ± 3.3)° after 2 postoperative years. In the PLIF group, IA increased from preoperative (8.25 ± 3.7)° to postoperative (9.56 ± 4.04)°. The IA was maintained at (9.03 ± 3.68)° after 2 years postoperatively. The IAs were significantly different at each postoperative follow-up between the ISDF and PLIF groups (*p* < 0.05).

The preoperative LL values of patients in both groups had no significant differences (ISDF: 31.62 ± 4.0°; PLIF: 31.54 ± 3.93°). However, the postoperative LL of the PLIF group was (33.61 ± 3.47)°, which was significantly different from the preoperative LL values (*p* < 0.05). Of note, the ISDF group had postoperative LL values of (31.56 ± 3.36)°, which was similar to the preoperative LL. After 2 years postoperatively, the LL was not significantly different between both groups (ISDF group: 31.47 ± 3.25°, PLIF group: 32.54 ± 3.31°).

The preoperative PDH score of the ISDF group was (0.72 ± 0.17) cm and that for PLIF was (0.74 ± 0.13) cm. The postoperative PDH increased to (0.84 ± 0.17) cm and (0.86 ± 0.17) cm for the ISDF and PLIF groups, respectively; After 2 postoperative years, it remained at (0.79 ± 0.16) cm and (0.84 ± 0.13) cm in the ISDF and PLIF groups, respectively. The PDH had no significant difference between both groups preoperatively or at each postoperative time point.

After 2 years postoperatively, interspinous bony fusion was clearly observed through sagittal CT reconstruction. The fusion rates were 79.41% (27/34) in the ISDF group and 85.71% (25/28) in the PLIF group, showing no significant difference in the lumbar fusion rates between both groups.

## Discussion

In the current study, the clinical outcomes between ISDF and PLIF were compared. The results demonstrated that ISDF surgery had a similar clinical efficacy as PLIF for octogenarians with LSS. The patients’ pain symptoms were remarkably relieved, and their quality-of-life and mobility capacity, were significantly improved. Moreover, compared to PLIF, ISDF is a relatively minimally invasive procedure, which can effectively reduce blood loss, shorten the operation time and hospitalization period. These results revealed that ISDF had a greater advantage over PLIF in octogenarians with LSS.

PLIF is considered as the “gold standard” in the surgical treatment of patients with LSS who are unresponsive for conservative treatment. However, patients who would undergo PLIF require good physical status, which is a tremendous challenge for octogenarian patients. Although advanced age is not considered as a contraindication for spinal surgery, the risk of surgery increases undoubtedly as age and comorbidities increase [26, 27]. In the recent years, ISDF has been used for lumbar degenerative disease because of its minimal invasiveness, which provides a new hope for octogenarian patients.

Previous studies have reported interspinous stabilization device can effectively alleviate pain and improve the quality of life[28]. Zheng et al. reported an 8-year follow-up study to compare the clinical efficacy between Coflex and PLIF in lumbar degenerative diseases[29]. The results indicated that Coflex can effectively improve VAS and ODI as compared with PLIF. Yuan et al. also conducted a 5-year follow-up retrospective study and reported no significant difference in terms of the improvement of ODI and VAS between the Coflex group and the PLIF group[30]. In our current study, similar results can be obtained. ISDF device provided similar VAS, ODI and JOA improvement compared to PLIF as a kind of fused interspinous stabilization device. In the clinical series studies, Falowski et al. demonstrated satisfying clinical efficacy of ISDF for LSS in a cohort of patients aged 71.8 years with blood loss < 50 ml[19]. Postacchini et al. also demonstrated the use of ISDF in LSS with lumbar spondylolisthesis with a good clinical efficacy in most patients without significant slipping progression after a 2-year follow-up[20]. In this study, we found a good clinical efficacy of ISDF in the octogenarians LSS patients.

In the radiological evaluation, the IA of the surgical segment significantly decreased and the PDH significantly increased in the ISDF group. Wei et al. also reported similar outcomes after the implantation of the ISDF device[17]. Some studies reported that Coflex can increase the PDH of the implanted segment after surgery. However, during the follow-up time, the correction PDH occurred loss[29, 31]. This may be related with different design principles. Even though the IA of the surgical segment significantly decreased in the ISDF group, the overall LL angle showed no change. Therefore, the single-segment implantation of the ISDF device did not influence the overall lumbar alignment. According to the literature, PLIF can effectively increase the PDH and IA of the fused level and improve LL through the implantation of the intervertebral cage and the correction of screw-rod[32, 33]. In our study, segmental IA, LL and PDH were slightly increased in the PLIF group. However, these corrections are closely related to the operative procedures, such as intervertebral fusion cage types and positions, rod curvature, and longitudinal compression[34].

Interspinous stabilization surgery is a relatively minimally invasive procedure, which can effectively reduce blood loss and shorten the operation time and hospitalization period, compared to PLIF[29]. In our study, only the partial lamina and interspinous ligament are resected in ISDF, which reduces the destruction of the spinal anatomical structures. However, PLIF requires extensive paravertebral muscle dissection, pedicle screw instrumentation, lamina resection, spinal canal exposure, and disc excision. Therefore, ISDF has less trauma than PLIF and is more suitable for octogenarian patients.

Spinal fusion surgeries were traditionally performed under general anesthesia, which requires a higher cardiopulmonary function. Most senior patients undertook a higher anesthesia risk due to a lack of physiological reserve capacity[35]. The patients in our study were octogenarians with all kinds of comorbidities. The CCI was 1.90 ± 0.76 and ASA score was 2.81 ± 0.40. All ISDF surgeries were performed under spinal anesthesia. Compared with general anesthesia, spinal anesthesia can reduce the influence on hemodynamic profiles, less dependence on postoperative pain medications, and lower urinary retention and nausea rates[36]. Therefore, ISDF can provide a chance of surgical procedure for octogenarian patients who are intolerant to general anesthesia as well as reduce perioperative complications.

The complication rate is an important factor for evaluating surgical safety. Puvanesarajah et al. assessed the complications of lumbar fusion surgery in octogenarian patients and concluded a high postoperative complication rate of 45.6%[37]. Raffo et al. also reported the incidence of major perioperative complications as 20% in octogenarian patients[25]. In this study, the complication rate in the PLIF group was 28.57% (8/28), while that of the ISDF group was 5.88% (2/34). Compared with PLIF, ISDF surgery can effectively reduce the incidence of perioperative complications. It seems logical that as the invasiveness of the surgical procedure reduces to minimal levels, the influence of comorbidity on the complication rates also reduces[25].

Successful lumbar fusion is an important factor that guarantees long-term surgical efficacy. Although many studies confirmed a high interbody fusion rate of PLIF, few have reported the results of interspinous fusion results. Karahalios et al. confirmed interspinous process fusion through radiological images in the research of intervertebral interbody fusion fixed with an ISDF device[38]. Franco reported an 84% interspinous fusion rate in a prospective study that involved the use of ISDF alone implant for degenerative spondylolisthesis[20]. In this study, an obvious bone bridge connection was observed in the sagittal CT image obtained 2 years after surgery in the ISDF group. The interspinous process fusion rate reached 79.41%, which was not significantly different from the 85.71% for PLIF. Therefore, interspinous process fusion can be achieved in ISDF surgery, which guarantees long-term surgical efficacy.

There are several limitations in our study. First, this is a retrospective study where patients selection was not random. Second, the sample size was small and with a short interval for follow up. Therefore, it is necessary to perform a prospective randomized control study with a larger sample size and longer follow-up time to improve the quality.

## Conclusion

ISDF surgery is a viable method for octogenarian patients with LSS that provides a similar clinical efficacy, shorter operative time, less blood loss, shorter hospital stay time, and fewer complications, compared to the PLIF surgery.

## Data Availability

Related data and materials can be obtained.
